# Single-Micelle-Templated Synthesis of Hollow Barium Carbonate Nanoparticle for Drug Delivery

**DOI:** 10.3390/polym15071739

**Published:** 2023-03-31

**Authors:** Bishnu Prasad Bastakoti, Nischal Bhattarai, Moses D. Ashie, Felix Tettey, Shin-ichi Yusa, Kenichi Nakashima

**Affiliations:** 1 Department of Chemistry, North Carolina A & T State University, 1601 E Market St, Greensboro, NC 27411, USA; 2 Western Guilford High School, 409 Friendway Rd, Greensboro, NC 27410, USA; 3 Department of Industrial and Systems Engineering, North Carolina A & T State University, 1601 E Market St, Greensboro, NC 27411, USA; 4 Department of Applied Chemistry, University of Hyogo, 2167 Shosha, Himeji 671-2280, Japan; 5 Department of Chemistry, Faculty of Science and Engineering, Saga University, 1 Honjo-machi, Saga 840-8502, Japan

**Keywords:** block copolymer, barium carbonate, hollow particles, drug delivery, micelles

## Abstract

A laboratory-synthesized triblock copolymer poly(ethylene oxide-*b*-acrylic acid-*b*-styrene) (PEG-PAA-PS) was used as a template to synthesize hollow BaCO_3_ nanoparticles (BC-NPs). The triblock copolymer was synthesized using reversible addition–fragmentation chain transfer radical polymerization. The triblock copolymer has a molecular weight of 1.88 × 10^4^ g/mol. Transmission electron microscopy measurements confirm the formation of spherical micelles with a PEG corona, PAA shell, and PS core in an aqueous solution. Furthermore, the dynamic light scattering experiment revealed the electrostatic interaction of Ba^2+^ ions with an anionic poly(acrylic acid) block of the micelles. The controlled precipitation of BaCO_3_ around spherical polymeric micelles followed by calcination allows for the synthesis of hollow BC-NPs with cavity diameters of 15 nm and a shell thickness of 5 nm. The encapsulation and release of methotrexate from hollow BC-NPs at pH 7.4 was studied. The cell viability experiments indicate the possibility of BC-NPs maintaining biocompatibility for a prolonged time.

## 1. Introduction

BaCO_3_ has attracted much research recently due to its many essential applications in producing pigments, catalysts, optical glass, and electric condensers [1,2,3,4,5,6,7]. Moreover, it is a precursor for producing magnetic ferroelectric, superconductors, and ceramic materials [8]. They are typically synthesized using the precipitation method [9,10,11]. Citric acid was used as a stabilizer to synthesize the BaCO_3_ nanoparticles (BC-NPs). The primary function of citric acid is to provide a polymeric network to hinder cations’ mobility, which reacts with Ba^2+^ ions to form a metal ion–citric acid complex. The complex is a platform for the crystallization of BC-NPs. The pure orthorhombic crystal was obtained after calcining at 450 °C. The crystallite sizes increased with an increase in the calcination temperature [12]. Nagajyothi et al. synthesized BC-NPs in an aqueous extract of mango seed. The sub-20 nm mixed (spherical and triangular) structured nanoparticles were used to treat cervical carcinoma [13]. BaCO_3_ is also used in tuning the optical properties of rare earth materials, which have wide applications in biomaterials, lasers, light-emitting diodes, and optical communication. It is considered as one of the favored host materials for lanthanide doping [14,15]. It brings new physical and chemical stability, high transparency, and good moisture resistance properties. Eu^3+^-doped BC-NPs were synthesized via the auto-combustion method [16]. The addition of the lanthanide ions did not change the structure and crystallinity; however, the optical properties of the composite were significantly improved.

Among the other morphologies, hollow nanostructures have attracted recent research interest due to their large pore volume, low mass density, and high surface-to-volume ratio compared to their solid counterparts [17,18,19]. Especially in bioapplication, the hollow void encapsulates the drug molecules and releases them when necessary [20]. The porous hollow shell is a diffusion barrier for the controlled release of payloads. Inorganic hollow nanoparticles are synthesized via various methods, such as soft templating, hard templating, and template-free methods [21,22]. The use of polymeric micelles as a template has proven to be a successful route toward synthesizing hollow structures with controllable size and morphology. We used a single-micelle templating method to synthesize the sub-50 nm hollow nanoparticles of metal, metal oxides, and metal phosphate [23,24,25]. In the recent report, a positively charged spherical micelle of poly(ethylene oxide-vinyl pyridine-styrene) (PEO-PVP-PS) block copolymer interacts with positively charged metal sources through a negatively charged phosphate ions bridge. After removing the polymer template via calcination, the hollow nanoparticles of nickel phosphate were obtained [26]. The dimension of the hollow void and shell thickness were easily tuned by controlling the molecular weight of the core and shell of the block copolymer [23,27].

**Figure 1:**
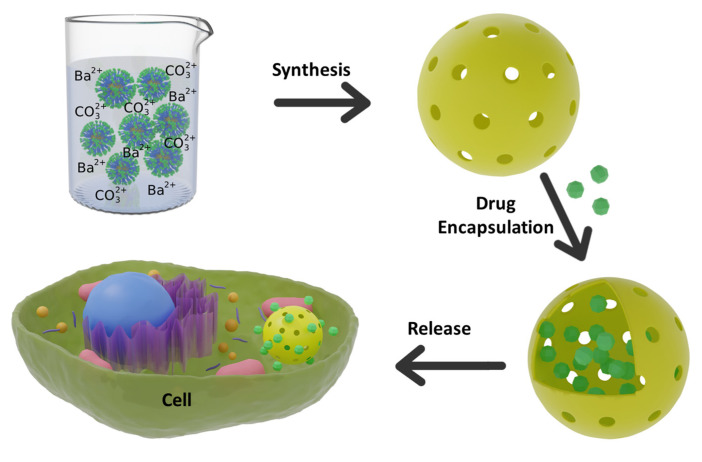
Synthesis of hollow BaCO_3_ nanoparticle and use of it as a drug carrier.

The synthesis of BaCO_3_ nanoparticles with the hollow void is always difficult due to the fast precipitation reaction between Ba^2+^_(aq)_ ions and CO_3_^2−^_(aq)_ ions. Here, we designed a polymer so that polymer micelles stabilize the BC-NPs. The reversible addition–fragmentation chain transfer (RAFT) radical polymerization was used to synthesize a triblock copolymer poly(ethylene oxide-*b*-acrylic acid-*b*-styrene) (PEG-PAA-PS). Ba^2+^ ions interact with the polyacrylic acid block to form a chelating complex that undergoes a selective precipitation reaction with carbonate on the PAA/Ba^2+^ ions shell. The interaction of metal ions with the polymer, the controlled precipitation of BaCO_3_, and the formation of hollow structures were characterized by various techniques, including dynamic light scattering (DLS), Fourier-transform infrared (FTIR) spectroscopy, thermogravimetric analysis (TGA), transmission electron microscopy (TEM), and X-ray diffraction (XRD). The calcination of BaCO_3_/polymer composites burns up the polymer, leaving the hollow void to encapsulate drug molecules and the porous shell as a diffusion barrier for controlled release (Figure 1). Finally, a cell proliferation assay was performed to check the biocompatibility of the nanoparticles to be used as drug carriers. As far as we have noticed, this is the first report on synthesizing sub-50 nm BC-NPs being used in drug delivery to date.

## 2. Materials and Methods

### 2.1. Materials

**Figure 2:**
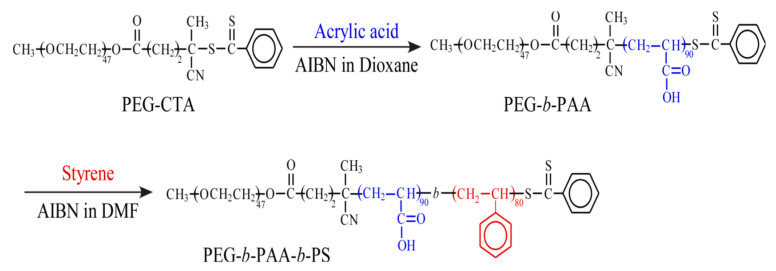
Synthesis of PEG-PAA-PS block copolymer via RAFT-controlled radical polymerization.

Poly(ethylene glycol) 4-cyano-4-(phenylcarbonothioylthio)pentanoate (PEG macro-CTA, average molecular weight = 2.00 × 10^3^ g/mol) from Aldrich (Tokyo, Japan) was used without further purification. 2.2′-Azobis(isobutyronitrile) (AIBN, >98%) from Wako pure chemical (Osaka, Japan) was recrystallized from methanol. Acrylic acid (AA, >99.0%), *N, N*-dimethylformamide (DMF, >99.5%), and 1,4-dioxane (>99.0%) from Tokyo Industry (Tokyo, Japan) were dried over 4 Å molecular sieves and distilled under reduced pressure. Styrene was washed with an aqueous alkaline solution and distilled from calcium hydride under reduced pressure. PEG-PAA-PS was synthesized (Figure 2) using RAFT polymerization as previously reported [25]. Na_2_CO_3_, BaCl_2_·2H_2_O, and methotrexate were purchased from Thermo Scientific (Waltham, MA, USA). A 0.1 M tris buffer (solution of pH 7.4, EMD millipore corp.) was used as release media. Dulbecco’s modified Eagle’s medium (DMEM) and Dulbecco’s phosphate-buffered saline (DPBS) were obtained from Life Technologies (Grand Island, NY, USA). Alamar Blue, lactate dehydrogenase (LDH) assay kit and every other materials not otherwise specified were purchased from Thermo Scientific.

### 2.2. Synthesis of Block Copolymer

First, we prepared PEG-PAA diblock copolymer as follows. PEG macro-CTA (475 mg, 0.2 mmol, *M*_n_(NMR) = 2.36 × 10^3^ g/mol), AA (2.18 g, 30.3 mmol), and AIBN (12.5 mg, 0.08 mmol,) were dissolved in 1,4-dioxane (95 mL) with a molar ratio of [AA]/[PEG macro-CTA]/[AIBN] = 151/1/0.38. The solution was purged with Ar gas for 30 min. Polymerization was carried out at 60 °C for 40 h under Ar gas. After polymerization, the obtained polymer was purified by dialysis against pure water for two days. The diblock copolymer (PEG-PAA) was recovered by a freeze-drying technique (1.7 g, 64.0%). Number-average degree of polymerization (DP) of the PAA block was estimated from ^1^H NMR spectrum in DMSO-*d*_6_ to be 90 (Figure S1). Number-average molecular weight (*M*_n_(NMR)) for PEG-PAA was 8.85 × 10^3^ g/mol. *M*_n_(GPC) and molecular weight distribution (*M*_w_/*M*_n_) were 1.53 × 10^4^ g/mol and 1.31, respectively (Figure S2), estimated from gel permeation chromatography (GPC). Styrene (10.4 g, 0.100 mol), AIBN (8.23 mg, 0.05 mmol), and PEG-PAA (1.11 g, 0.13 mmol, *M*_n_(theo) = 8.85 × 10^3^ g/mol) were dissolved in DMF (100 mL) with a molar ratio of [styrene]/[PEG-PAA]/[AIBN] = 798/1/0.40. The solution was degassed by purging with Ar gas for 30 min. The polymerization was carried out at 60 °C for 24 h. The conversion of styrene was 20.6%, determined from ^1^H NMR for the polymerization mixture. The polymerization mixture was poured into a large excess of ethyl acetate. The precipitate was dialyzed against tetrahydrofuran for three days and pure water for a day. The triblock copolymer (PEG-PAA-PS) was recovered by a freeze-drying technique (2.06 g, 19.8%). DP of the PS block was 80 as estimated by ^1^H NMR in DMSO-*d*_6_ at 100 °C. *M*_n_(NMR) for the triblock copolymer was 1.88 × 10^4^ g/mol (Figure S3). We could not perform GPC measurements for PEG-PAA-PS because the polymer can molecularly dissolve only in DMSO at high temperatures.

### 2.3. Preparation of Polymeric Micelle and Synthesis of BaCO_3_ Nanoparticles

A total of 20 mg of the polymer was dissolved in 10 mL of tetrahydrofuran. The solution was sonicated for 20 min and magnetically stirred for 12 h. The solution was dialyzed against water to prepare an aqueous solution of the polymer. The final concentration of polymer was made to be 0.1 g/L^−1^ at pH 9. A total of 20 mg barium chloride was added to 20 mL of a micelle solution (0.1 g/L^−1^) and stirred for 1 h. A total of 8.8 mg of sodium carbonate was added to the mixture, leaving 2 h for precipitation reaction. The BaCO_3_/polymer composite particles were recovered by centrifugation. The composite particles were thoroughly washed with deionized water and dried in an oven at 50 °C before calcination at 550 °C for 3 h at a ramping rate of 2 °C min^−1^.

### 2.4. Characterization

The kinetics and progress of the polymerization reaction were studied using a Bruker DRX-500 nuclear magnetic resonance (NMR) spectrometer operating at 500 MHz and gas permeation chromatography (GPC). A refractive index (RI) detector equipped with a Shodex GF-7M HQ column working at 40 °C and a flow rate of 0.6 mL/min was used in the GPC measurement of PEG-PAA. A phosphate buffer (pH 8) containing 10 vol % acetonitrile was used as the eluent. The instrument was calibrated using the sodium poly(styrene sulfonate) standard. The dynamic light scattering measurements were carried out using an Otsuka ELS Z zeta potential and particle analyzer. All of the measurements were carried out at 25 °C and filtered through a 0.4 µm cellulose filter. The concentration and pH of the solution was maintained at 0.1 g/L and 9, respectively. The hydrodynamic diameter (*D_h_*) was calculated using Stokes–Einstein equation (*D_h_ = k_B_T*/3π*η*D). Here, *k_B_* is the Boltzmann constant, *T* is the absolute temperature, D is the diffusion coefficient, and η is the solvent viscosity. The zeta potential (*ζ*) was calculated from the Smolichowski equation (*µ_E_ = ζε*/*η*). *µ_E_* is electrophoresis mobility and *ε* is the permittivity of the solvent. The TEM measurements were carried out using a JEOL JEM-1210 electron microscope at an accelerating voltage of 80 kV. The hollow barium carbonate nanoparticles were dispersed into ethanol and cast on the copper grid for TEM measurement. For polymeric micelles, phosphotungstic acid was used as a contrasting agent. The crystallinity and phase identification of the hollow nanoparticles was carried out using a Shimadzu-630D X-ray diffractometer at 40 kV, 30 mA, and with CuKα radiation (1.5406 Å). A simultaneous thermogravimetric analysis (TGA) and a differential thermal analysis (DTA) were carried out using a SEIKO-6300 TG/DTA instrument at a heating rate of 10 °C min^−1^ in air. FTIR data were collected from the IR Tracer-100 in attenuated total reflection mode (Shimadzu, Kyoto, Japan).

### 2.5. Drug Release

A total of 50 mg of BC-NPs was dispersed into 2.5 mL of 4 mM methotrexate drug solution. The solution was sonicated for 60 min and magnetically stirred for 24 h in the dark for equilibration. A dialysis cassette was wetted for 2 min before sample injection. The equilibrated drug solution was injected into the cassette, ensuring no air bubbles. The cassette was diagonally placed in a beaker containing 100 mL of tris buffer solution of pH 7.4. The buffer (release medium) was stirred at a constant rate of 100 rpm at room temperature. A 3 mL solution from the beaker was sampled, and the absorbance of methotrexate was taken. The sample solution was returned to vessels after every reading. In the controlled experiment, 2.5 mL of 4 mM methotrexate drug solution was injected into the cassette and the release phenomenon without using nanoparticles was studied.

### 2.6. Cell Preparation

NIH-3T3 cells (a mouse fibroblast cell line, ATCC 1658) were maintained in standard DMEM supplemented with 10% FBS, 1% pen strip, and 0.12% insulin. The cells were cultured in 75 cm^2^ tissue culture flasks at 37 °C in a 5% CO_2_ humidified environment. Cells were trypsinized using 0.25% trypsin/EDTA at 80% confluence. Trypsinized cells formed pellets when centrifuged and were re-suspended in fresh media at the required cell density. The experiment utilized passage number eight cells.

### 2.7. Toxicity Study

The BC-NPs were first exposed under UV light for 12 h and were suspended in sterile deionized water by sonication for 30 min. To make different exposure solutions (e.g., 0, 50, 100, and 500 μg mL^−1^), the BC-NPs solution was added to the DMEM medium containing 10% FBS and was thoroughly mixed via vortexing. The BC-NPs solutions were labeled as BC-0, BC-50, BC-100, and BC-500. The BC-0 sample, which has no BC-NPs, represents the control sample, whereas BC-50 to 500 represents the different amounts of NPs used. The cells were treated with 1 mL of the different concentrations of BC-NPs according to a previous publication [28]. Cells treated with the different concentrations of BC-NPs solutions were cultured at 37 °C in a 5% CO_2_ humidified environment. At specific time points (24 h, 48 h, and 72 h), the supernatant of the cell–particle medium was collected and stored for cytotoxicity study. The cytotoxicity effect of the BC-NPs on the fibroblast cells was determined using the Pierce lactate dehydrogenase (LDH) cytotoxicity assay kit as described in our earlier publication [29,30]. Briefly, 50 μL of each stored sample was transferred to a 96-well flat-bottom plate and triplicated. Next, 50 μL of the reaction mixture generated from the protocol was added to each sample well, and the plate was kept in the dark for 30 min at room temperature. The positive and negative controls were established based on the manufacturer’s kit protocol. The reaction mixture kept in the dark was stopped by adding 50 μL of stop solution to each sample well and mixed by gentle tapping. The absorbance of the assay solution in the well plate was measured at 490 nm and 680 nm using a CLARIOstar^®^ multi-mode plate reader (BMG LABTECH Inc., Cary, NC, USA). The cytotoxicity was then calculated using the following equation:

(1) $$\mathrm{Cytotoxicity}=\frac{\mathrm{OD}\;\mathrm{at}\;490\;\mathrm{nm}\;\mathrm{test}\;\mathrm{sample}-\mathrm{OD}\;\mathrm{at}\;490\;\mathrm{nm}\;\mathrm{negative}\;\mathrm{control}}{\mathrm{OD}\;\mathrm{at}\;490\;\mathrm{nm}\;\mathrm{positive}\;\mathrm{sample}-\mathrm{OD}\;\mathrm{at}\;490\;\mathrm{nm}\;\mathrm{negative}\;\mathrm{control}}\times 100\%$$

where OD is absorbance.

### 2.8. Cell Viability Study

3T3 cells were observed with an Alamar Blue (AB) colorimetric assay according to the manufacturer’s standard protocol [31]. For this assay, the medium was removed from the cells treated with BC-NPs after being washed twice with PBS and then incubated for four hours with 5% (*v*/*v*) AB reagent in the respective culture medium (50 μL of AB reagent in 1 mL culture medium). Assay solutions were transferred to fresh plates, and multiple aliquots were taken to measure fluorescence on a microplate reader (CLARIOstar Plus, BMG LABTECH Inc., Cary, NC, USA) with excitation at 530 nm and emission at 590 nm. Cell viability was calculated using the following equation:

(2) $$\mathrm{Cell}\;\mathrm{Viability}=\frac{\mathrm{Fluorescence}\;\mathrm{of}\;\mathrm{the}\;\mathrm{sample}-\mathrm{Fluorescence}\;\mathrm{of}\;\mathrm{the}\;\mathrm{blank}}{\mathrm{Fluorescence}\;\mathrm{of}\;\mathrm{the}\;\mathrm{control}-\mathrm{Fluorescence}\;\mathrm{of}\;\mathrm{the}\;\mathrm{blank}}\times 100\%$$

All results were expressed as mean ± SD. Data were analyzed for significance with OriginPro Version 2023 software (Origin Lab, Northampton, MA, USA).

### 2.9. Fluorescence Imaging and Analysis

Fluorescence imaging of the 3T3 fibroblast cells with BC-NPs was completed as previously reported [29]. Briefly, the excess medium was removed, and samples were washed twice with DPBS and then stained with 15 μL of acridine orange (AO) and propidium iodide (PI) nucleic acid binding dyes (Nexcelom Bioscience, Lawrence, MA, USA) and incubated at 37 °C for 10 min. Images were photographed under an Olympus IX83 microscope using Olympus cell Sens Dimension software (Olympus Corporation, Shinjuku, Tokyo, Japan).

## 3. Results and Discussion

**Figure 3:**
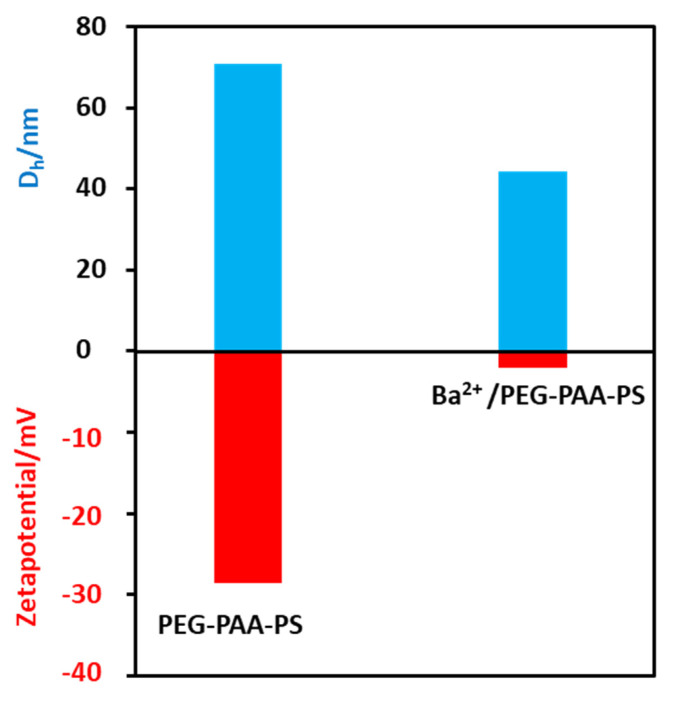
Hydrodynamic diameter (*D_h_*) and zeta potential of pure polymeric micelles and micelles composites.

**Figure 4:**
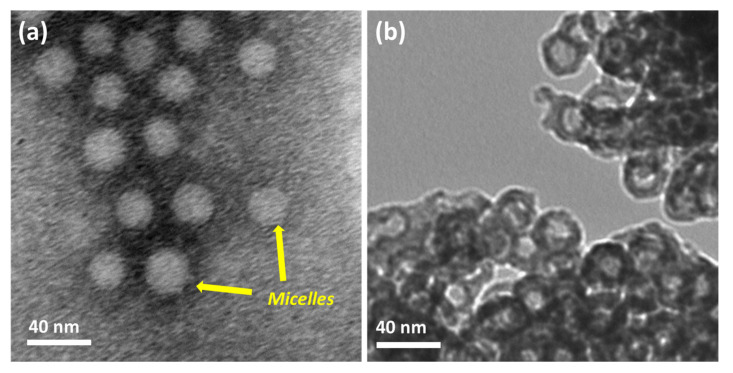
TEM mages of (**a**) polymeric micelles and (**b**) hollow BaCO_3_ nanoparticles.

The polymer was first dissolved in THF and collected in water by dialysis. The exchange of THF with water makes the solution slightly turbid, indicating the formation of micelles. The Tyndall effects on the polymer solution confirm to the formation of colloidal nanoaggregates (Figure S4). The dynamic light scattering experiment shows that the hydrodynamic diameter (*D_h_*) of the micelles is 74 nm (Figure 3). The zeta potential of the polymer solution at pH 9 is −28 mV. We believe that the polyacrylic acid chains exhibit fully extended conformation as the solution pH is higher than the p*K*_a_ (4.5) value of acrylic acid [32]. The advantage of these micelles (negatively charged) is that the positively charged metal ions strongly bind electrostatically. The addition of Ba^2+^ ions turns the solution slightly more turbid, indicating an increased hydrophobicity of Ba^2+^/PEG-PAA-PS nanoaggregates (Figure S4). The *D_h_* of Ba^2+^/PEG-PAA-PS decreases to 43 nm, suggesting a decrease in electrostatic repulsion among PAA units due to the neutralization of the negative charge of PAA with Ba^2+^ ions. The increase in the zeta potential from −28 mV to −3 mV after the addition of metal ions further confirms the masking of the negative charge of micelles by positively charged metal ions. The block copolymer is expected to form uniform spherical micelles in an aqueous solution with the PEG corona, PAA shell, and PS core. After staining with phosphotungstic acid, nanoaggregates were observed by TEM, and spherical micelles were obtained. This provides concrete evidence of the formation of micelles in water. The white sphere indicates a negatively stained PS core (Figure 4a). The micelles’ size (30 nm) from TEM is much smaller than that from DLS (74 nm). This discrepancy is due to the different modes of measurement. DLS is a solution-based measurement. The water around the micelles helps to expand their size. The hydrophilic parts (PEG and deprotonated PAA) have a fully extended form. However, TEM measurement is performed in dry and high vacuum conditions. The micelles are dry and shrunken, which makes the size obtained from TEM always smaller than that of DLS.

Polymeric micelles are widely used as a template and structure-directing agent for synthesizing hollow nanoparticles with controllable dimensions. Nakashima et al. synthesized silica nanosphere templating on PEO-PVP-PS block copolymer [33]. The controlled polymerization of silica precursors with the micelles template allows for fabricating hollow nanospheres with different shell thicknesses and hollow voids. The addition of Ba^2+^ ions into a micelles solution forms a chelating complex with PAA blocks. One of the most exciting applications of the PEG-PAA-PS micelles in this study is a template for synthesizing inorganic hollow particles of BaCO_3_. Ba^2+^ ions first interact with micelles electrostatically.

**Figure 5:**
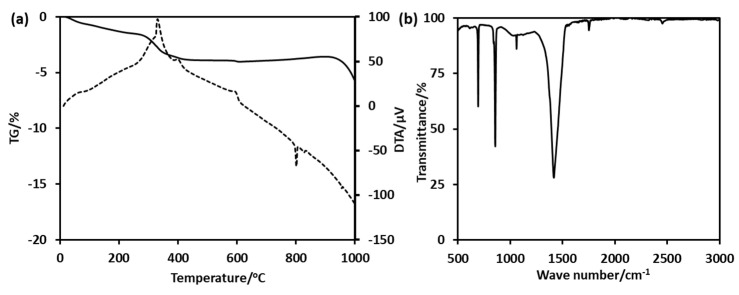
(**a**) Thermogravimetric analysis (TGA) results of BaCO_3_/PEG-PAA-PS nanocomposites and (**b**) Fourier transform infrared (FTIR) spectrum of hollow BaCO_3_ nanoparticles.

The precipitation of BaCO_3_ occurs around the Ba^2+^/PEG-PAA-PS nanoaggregates to form BaCO_3_/PEG-PAA-PS nanocomposites. The removal of the polymeric template by calcination led to the formation of hollow BC-NPs (Figure 4b). The cavity diameter is approximately 15 nm, with a thickness of 5 nm. The void diameter is smaller than the size of the PS core. This happens because the materials shrink during calcination at high temperatures. It is not easy to synthesize the sub-50 nm hollow nanoparticles due to the extremely fast kinetics of the formation of BaCO_3_. Salvatori et al. studied the nucleation and growth rate of BaCO_3_ [34]. The particles formed are generally micron-sized. Specific additives, such as block copolymers, strongly influence nucleation, growth, and self-organization [35]. The use of a triblock copolymer made the method easy and opened the avenue to synthesizing hollow structures with controllable dimensions. The slight weight loss at the initial part of the thermogravimetric analysis curves is due to the desorption of absorbed water molecules (Figure 5a). The strong exothermic peaks on the differential thermal analysis (DTA) curves around 400 °C show the thermal decomposition of the template polymer. The high carbon content and higher thermal stability of the polymer suppress the unwanted crystal growth at higher temperatures. The higher weight loss after 850 °C and a large endothermic peak on the DTA curve is possibly attributed to the decomposition of the BaCO_3_ crystal. FTIR confirmed the formation of BaCO_3_ nanoparticles (Figure 5b). The sharp peaks at 692 cm^−1^ and 856 cm^−1^ are the in-plane and out-of-plane bending of the CO_3_^2−^ ion. The 1415 cm^−1^ band corresponds to the asymmetric stretching mode of C-O, and the weak band at 1060 cm^−1^ is attributed to the symmetric C-O stretching vibration. The crystalline characteristics of the obtained BC-NPs were confirmed by XRD analysis. The sharp diffraction peaks (Figure S5) of the sample indicate that well-crystallized BC-NPs were obtained under the current synthetic conditions. No characteristic peaks due to impurities were detected, indicating that the BaCO_3_ nanoparticles are of high purity.

**Figure 6:**
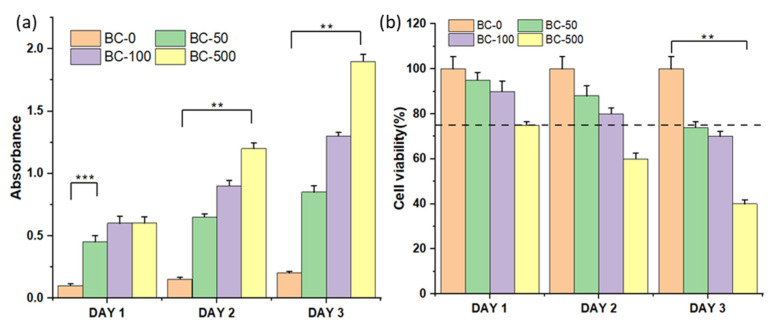
(**a**) Cytotoxicity graph and (**b**) cell viability graph of the BC-NPs with the 3T3 fibroblast cells (*n* = 3), where ** represents *p* < 0.01 and *** represents *p* < 0.001.

**Figure 7:**
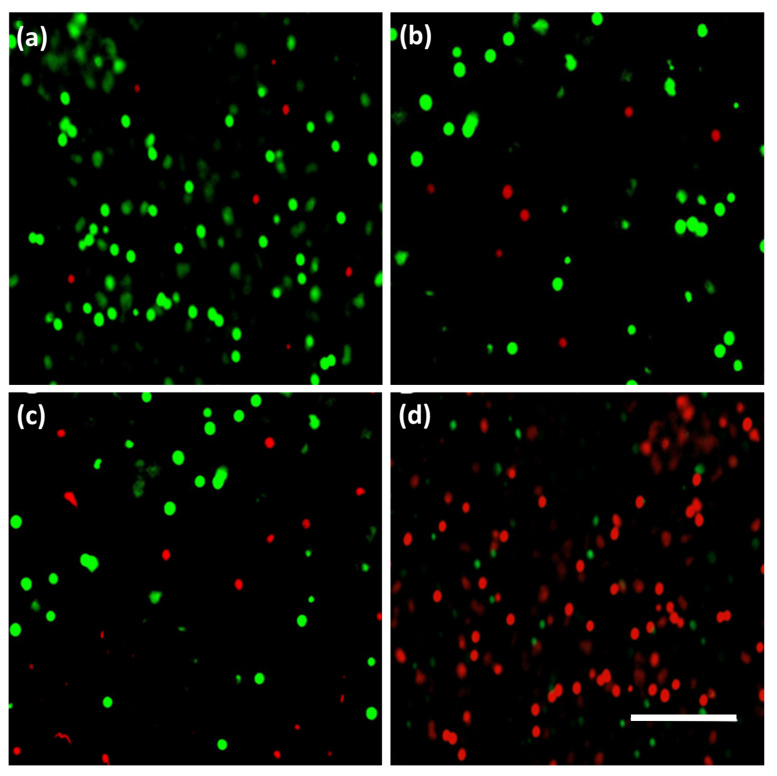
Fluorescence image showing live (green) and dead (red) cells of 3T3 fibroblast cells after exposure to different concentrations of BC-NPs at day 3. BC-0 (**a**), BC-50 (**b**), BC-100 (**c**), and BC-500 (**d**). All images were taken at 20× magnification. The scale bar is 100 µm.

The 3T3 fibroblast cells were treated with different concentrations of BC-NPs (BC-0, BC-50, BC-100, and BC-500) for up to three days, as shown in Figure 6. In evaluating the in vitro cytotoxic activity of BC-NPs, we observed no significant difference in LDH release among all of the tested samples on day 1. However, the highest induced BC-NPs (BC-500) showed a considerable amount of LDH release on day 3, which translates to its toxicity, and thus the substantial dead cells representation in the fluorescence image in Figure 7. The sample with the least BC-NPs (BC-50) resulted in the least LDH release overall. The cell proliferation assay using Alamar Blue (Figure 6b) shows that the cell viability remains relatively unchanged in all of the tested samples except for the sample with the concentration of nanoparticles increased to 500 μg mL^−1^, which showed cell viability deterioration on day 2 and more significantly on day 3. These findings are comparable with previously reported literature [28]. The cell viability graph indicates the possibility of BC-NPs maintaining biocompatibility after day 3, mainly for the BC-50 and possibly BC-100-induced samples. However, a perfect sample amount recommendation will be the BC-50, which satisfies cell compatibility even after day 3 and can further be evaluated and implemented in the future for biomedical applications.

The hollow nanoparticles have several advantages for drug delivery due to the high surface-to-volume ratio, porosity, and large void that can accommodate a large number of drug molecules to be delivered. Silica-based materials have been widely used as drug carriers. It has been reported that doxorubicin-loaded hollow nanoparticles were taken up by cancer cell lines and released the drug into the tumor. The growth rate was better reduced compared to the case where the free drug was introduced [36]. We investigated the release pattern of methotrexate from hollow BC-NPs at pH 7.4 using the dialysis method. First, the drug was encapsulated into the hollow nanoparticles and released from the hollow nanoparticles to the bulk aqueous phase inside the dialysis cassette. Then, it diffused to the aqueous phase outside the dialysis tube. The amount of the released drug was monitored outside of the dialysis membrane spectrophotometrically. The absorbance of methotrexate was recorded at 375 nm. It was observed that the drugs were released sustainably without burst release (Figure S6). We showed the drug release profile without using nanoparticles for comparison. In the first three hours, the release rate was similar for the hollow nanoparticles and the controlled experiment. This may be due to the loosely bound drug molecules on the surface of BaCO_3_ hollow nanoparticles. However, at the later stage, the drug molecules were released more slowly from the hollow nanoparticles than in the controlled condition. Hybridizing BaCO_3_ with other materials such as SiO_2_, TiO_2_, and Fe_2_O_3_ or functionalizing nanoparticles with stimuli responsive polymer would control the drug loading and release phenomena [37,38,39].

## 4. Conclusions

BaCO_3_ hollow nanoparticles were synthesized using a laboratory-designed triblock copolymer. RAFT-controlled radical polymerization allows for the synthesis of the block copolymer with the desired molecular weight. The triblock copolymer PEG-PAA-PS undergoes self-assembly to form spherical micelles with three chemically distinct domains in an aqueous solution. The hydrophobic PS core is a template used to generate a hollow void. The negatively charged PAA shell plays a vital role in arresting Ba^2+^ ions for the selective precipitation of BaCO_3_ on the shell region of the micelles. The hydrophilic PEG provides stability to the polymer–inorganic composite nanostructures after the precipitation reaction of BaCO_3_. The removal of the polymeric portion of the nanocomposites at high temperatures gives rise to hollow nanoparticles of BaCO_3_, proving that the polymeric system could be an excellent template for fabricating the hollow nanoparticles of inorganic materials undergoing a fast precipitation reaction. The in vitro release of methotrexate from the hollow particles shows that 64% of the drugs were released after 5.5 h. The encapsulated drug release was slower than the free drug molecules. However, the release kinetics are identical at the early stage, showing that drug molecules are loosely adsorbed on the surface of BaCO_3_ nanoparticles. Making composites or a suitable functionalization of BaCO_3_ nanoparticles would allow for the control of drug release kinetics. In addition, the obtained BC-NPs exhibit a very high biocompatibility, showing great promise for intracellular bio-applications in the future.

## Data Availability

Data will be made available on request.

## Supplementary Materials

The following supporting information can be downloaded at: https://www.mdpi.com/article/10.3390/polym15071739/s1, Figure S1. ^1^H NMR for PEG-PAA in DMSO-d_6_ at 100 °C. Figure S2. GPC elution curve for PEG-PAA using phosphate buffer as an eluent at 40 °C. Figure S3. ^1^H NMR for PEG-PAA-PS in DMSO-d_6_ at 100 °C. Figure S4: Tyndal effect showing the formation of colloidal particles: (**a**) PEG-PAA-PS polymer, (**b**) Ba^2+^/PEG-PAA-PS (**c**) BaCO_3_/PEG-PAA-PS aqueous solutions. Figure S5: XRD spectrum of hollow BaCO_3_ nanoparticles. Figure S6: Drug release profile from hollow BaCO_3_ nanoparticles. A_t_ and A_∞_ are the absorbance of the released drug at time t and infinity, respectively.
