# Supplementary material for: Single-Micelle-Templated Synthesis of Hollow Barium Carbonate Nanoparticle for Drug Delivery
Source: Polymers (Basel). 2023 Mar 31;15(7):1739. doi: 10.3390/polym15071739 (PMC10096637; doi:10.3390/polym15071739)
Supplement: Supplementary file 1 [file polymers-15-01739-s001.zip › polymers-2273037-SI.pdf]

Supporting Information

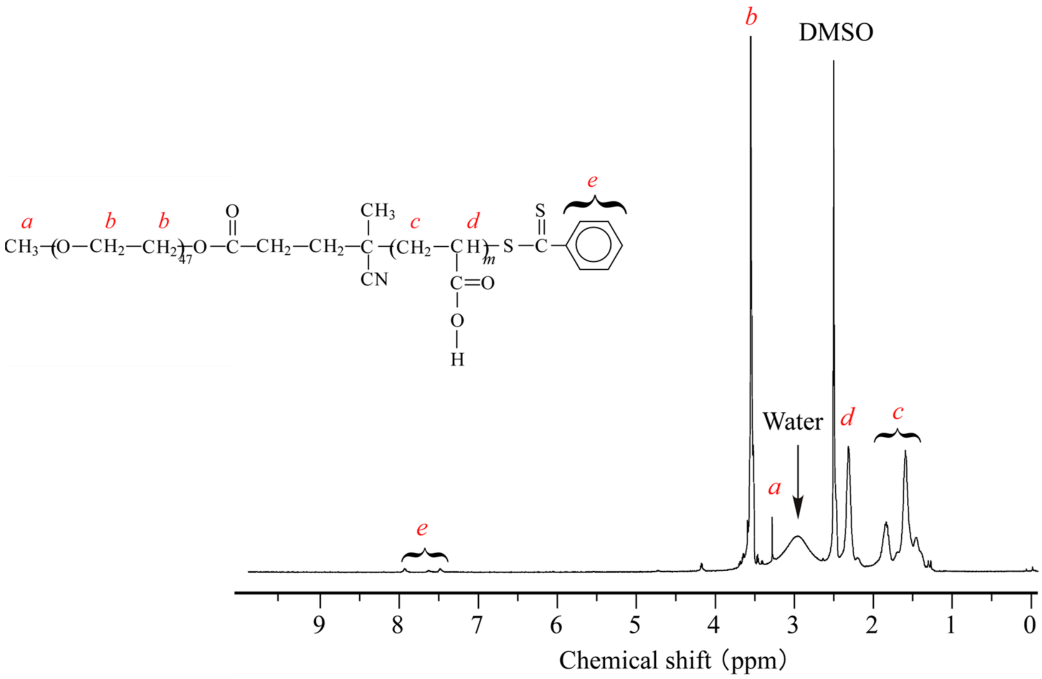

Figure S1. ^{1}H NMR for PEG-PAA in DMSO-d_6 at 100 °C.

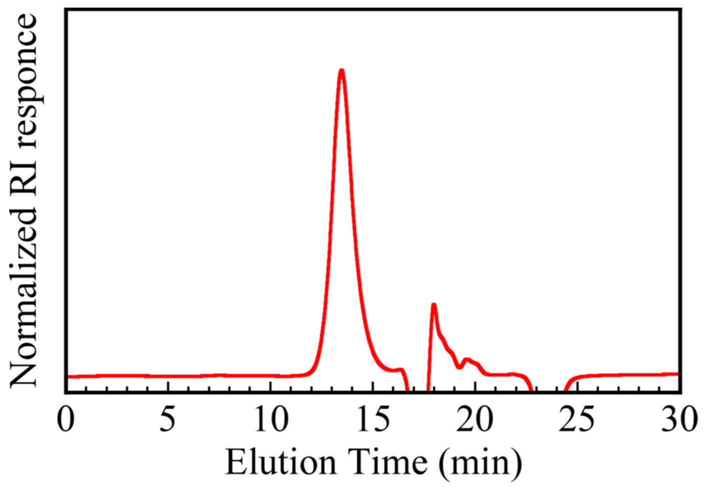

Figure S2. GPC elution curve for PEG-PAA using phosphate buffer as an eluent at 40 °C.

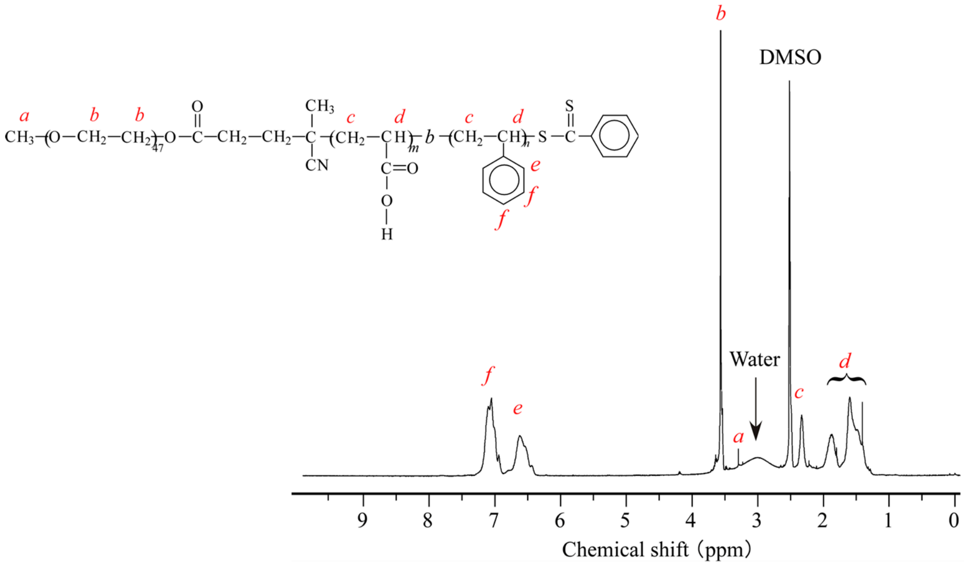

Figure S3. ^{1}H NMR for PEG-PAA-PS in DMSO-d_6 at 100 °C.

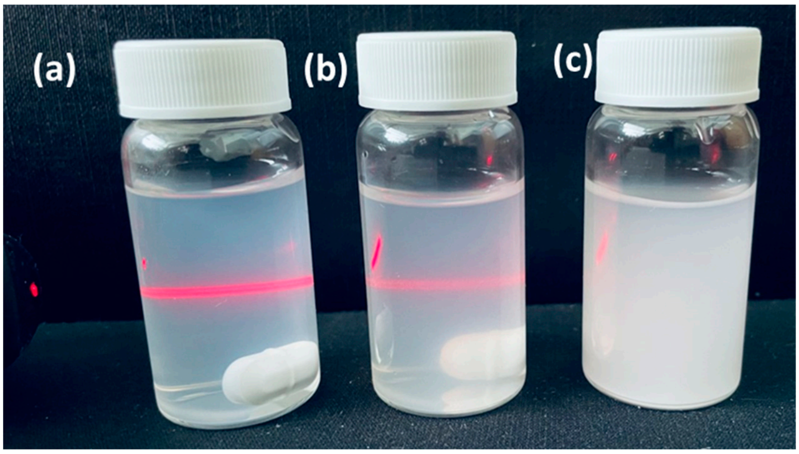

Figure S4. Tyndal effect showing formation of colloidal particles (a) PEG-PAA-PS polymer, (b) Ba^{2+}/PEG-PAA-PS (c) $BaCO_3$/PEG-PAA-PS aqueous solutions.

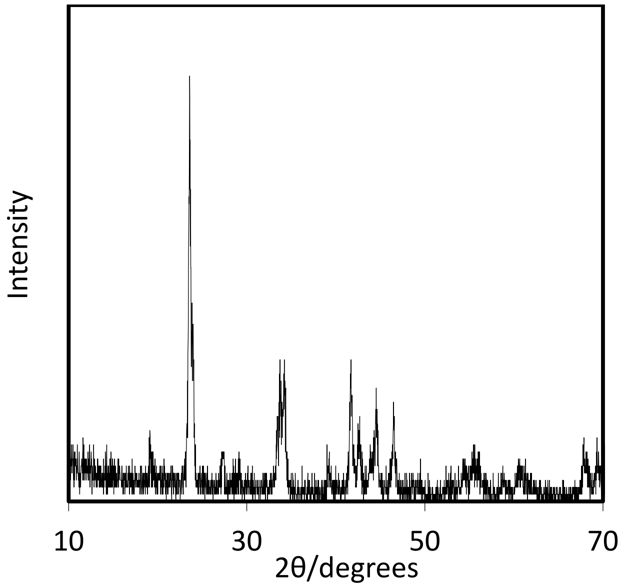

Figure S5. XRD spectrum of hollow $BaCO_3$ nanoparticles.

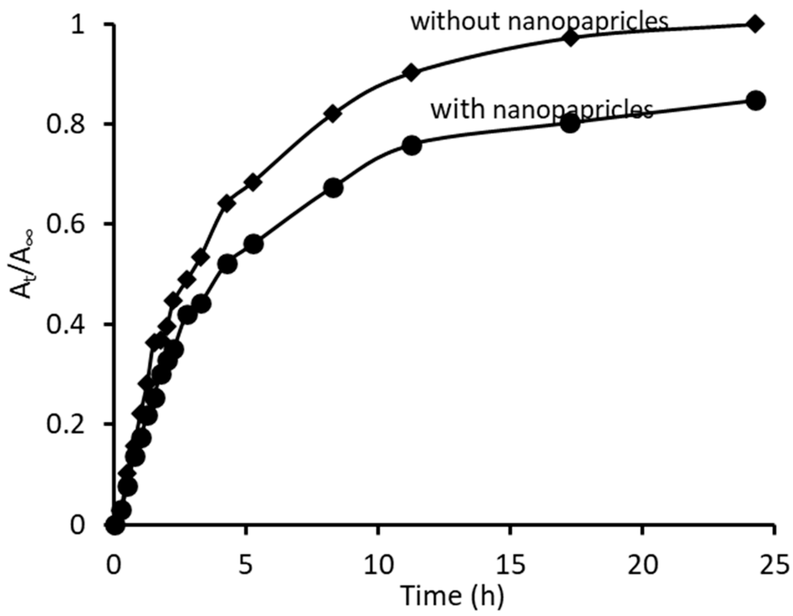

Figure S6. Drug release profile from hollow $BaCO_3$ nanoparticles. A_t and A_∞ are the absorbance of the released drug at time t and infinity, respectively.
